# Phylogenetic and Pathogenic Evidence Reveals Novel Host–Pathogen Interactions between Species of *Lasiodiplodia* and *Citrus latifolia* Dieback Disease in Southern Mexico

**DOI:** 10.3390/jof10070484

**Published:** 2024-07-14

**Authors:** Ricardo Santillán-Mendoza, Humberto Estrella-Maldonado, Lucero Marín-Oluarte, Cristian Matilde-Hernández, Gerardo Rodríguez-Alvarado, Sylvia P. Fernández-Pavía, Felipe R. Flores-de la Rosa

**Affiliations:** 1 Campo Experimental Ixtacuaco (CEIXTA), Centro de Investigación Regional Golfo Centro (CIRGOC), Instituto Nacional de Investigaciones Forestales, Agrícolas y Pecuarias (INIFAP), Km 4.5 Carretera Federal Martínez de la Torre-Tlapacoyan, Tlapacoyan 93600, Veracruz, Mexico; estrella.humberto@inifap.gob.mx (H.E.-M.); luceromarinoluarte@gmail.com (L.M.-O.); matilde.cristian@inifap.gob.mx (C.M.-H.); 2 Instituto de Investigaciones Agropecuarias y Forestales, Universidad Michoacana de San Nicolás de Hidalgo, Km 9.5 Carretera Morelia-Zinapécuaro, Tarímbaro 58880, Michoacán, Mexico; gra.labpv@gmail.com (G.R.-A.); patricia.pavia@umich.mx (S.P.F.-P.)

**Keywords:** Botryosphaeriaceae, *L. lignicola*, *L. mexicanensis*, Persian lime, ITS, *tef1-α*, *tub2*, *rpb2*

## Abstract

Mexico ranks second in the world for Persian lime (*Citrus latifolia*) exports, making it the principal citrus exporter within the national citrus industry, exporting over 600,000 tons per year. However, diseases are the main factor reducing production, resulting in significant economic losses. Among these diseases, fungal diseases like dieback, caused by species of *Lasiodiplodia*, are an emerging issue in Persian lime. Symptoms include gummosis, twig and branch dieback, cankers, the necrosis of bark and wood, fruit mummification, and tree decline. The aim of this study was to investigate the occurrence and pathogenicity of the fungal species associated with twig and branch dieback, cankers, and decline of Persian lime trees in southern Mexico, and to elucidate the current status of the *Lasiodiplodia* species causing the disease in Mexico. During June, July, and August of 2023, a total of the 9229 Persian lime trees were inspected across 230 hectares of Persian lime orchards in southern Mexico, and symptoms of the disease were detected in 48.78% of the trees. Branches from 30 of these Persian lime trees were collected. Fungal isolates were obtained, resulting in a collection of 40 strains. The isolates were characterized molecularly and phylogenetically through the partial regions of four loci: the internal transcribed spacer region (ITS), the β-tubulin gene (*tub2*), the translation elongation factor 1-alpha gene (*tef1-α*), and the DNA-directed RNA polymerase II second largest subunit (*rpb2*). Additionally, pathogenicity was assessed, successfully completing Koch’s postulates on both detached Persian lime branches and certified 18-month-old Persian lime plants. Through multilocus molecular phylogenetic identification, pathogenicity, and virulence tests, five species were identified as causal agents: *L. iraniensis*, *L. lignicola*, *L. mexicanensis*, *L. pseudotheobromae*, and *L. theobromae*. This study demonstrates that in southern Mexico, at least five species of the genus *Lasiodiplodia* are responsible for dieback in Persian lime. Additionally, this is the first report of *L. lignicola* and *L. mexicanensis* as causal agents of the disease in citrus, indicating novel host interactions between species of *Lasiodiplodia* and *C. latifolia*.

## 1. Introduction

Globally, citriculture is regarded the most important agricultural endeavor within fruit production, yielding over 150 million tons annually. Citrus fruits are cultivated on all five continents, with Asia contributing 51.89%, the Americas 29.39%, Africa 11.44%, Europe 6.88%, and Oceania 0.39% of the global production [1]. The leading producing nations are China, Brazil, India, Mexico, and the United States.

Mexico ranks as the fourth largest citrus producer worldwide, cultivating a total of 852,717 hectares of various *Citrus* species. These include sweet orange (*C. sinensis*), Persian lime (*C. latifolia*), key lime (*C. aurantifolia*), tangerine (*C. reticulata*), and grapefruit (*C. paradisi*), with a production exceeding 8.9 million tons, representing a production value over USD 3 billion [1,2]. Regarding Persian lime, Mexico produces over 1.5 million tons, with a production value exceeding USD 650 million. Mexico ranks second globally in export, with more than 600 thousand tons, mainly to North American, European, and Asian markets, being the primary supplier to the United States of America [1,2].

However, one of the principal factors limiting citrus production is disease caused by fungal pathogens. Notable among these diseases are anthracnose caused by the *Colletotrichum gloeosporioides* species complex [3], gummosis caused by *Phytophthora* spp. [4], dieback, and decline caused by species of the Botryosphaeriaceae family [5,6]. These microorganisms cause symptoms such as chlorosis, reduced growth and development, deficiencies in water and nutrient absorption, rot, necrosis, gummosis, cankers, branch dieback, and plant death, in some cases [6,7].

Members of the Botryosphaeriaceae family cause trunk and branch diseases, leading to significant production losses [8]. *Lasiodiplodia* is one of the most phytopathologically important genera within this family, currently comprising 48 species widely distributed around the world and found on a broad spectrum of hosts, including monocotyledonous, dicotyledonous, and gymnospermous plants [5,9,10,11]. Most *Lasiodiplodia* species are known as pathogens, causing various plant diseases like stem cankers, gummosis on stems and branches, shoot blight, and fruit rot [9,11,12,13]. Furthermore, they are frequently observed as endophytes and saprobes. Under abiotic stress conditions, they thrive in subtropical and tropical regions, affecting more than 1000 hosts [14,15,16].

Due to the similarity in the cultural and morphological characteristics of species of the Botryosphaeriaceae, molecular and phylogenetic characterizations are essential for distinguishing the species [17,18]. Zhang et al. (2021) [10] found that species of the genera *Botryosphaeria*, *Diplodia*, *Dothiorella*, and *Pseudofusicoccum* can be phylogenetically separated using ITS, *tef1-α*, and *tub2*, while species of the genera *Lasiodiplodia*, *Neofusicoccum*, *Neoscytalidium*, *Phaeobotryon*, and *Saccharata* require ITS, LSU, *tef1-α*, *tub2*, and *rpb2*. However, it was recently determined that for accurate identification of *Lasiodiplodia* species, the combination of four loci—ITS, *tef1-α*, *tub2*, and *rpb2*—is necessary for reliable resolution, which was established among the possible multilocus combinations with SSU, LSU, ITS, *tef1-α*, *tub2*, and *rpb2* [15].

*Lasiodiplodia* species are the primary etiological agents of citrus dieback and have been reported in various countries worldwide. For example, in Algeria, *L. mediterranea* and *L. mitidjana* were reported as the etiological agents of dead shoots, defoliation, cankers, wood necrosis, and dieback in *C. sinensis* [19]; in Brazil, *L. caatinguensis* and *L. theobromae* have been associated with gummosis and dieback [20,21]; in China, a study on seven citrus species (*C. grandis*, *C. limon*, *C. maxima*, *C. paradisi*, *C. reticulata*, *C. sinensis*, and *C. unshiu*) found that *L. citricola*, *L. guilinensis*, *L. huangyanensis*, *L. iraniensis*, *L. linhaiensis*, *L. microconidia*, *L. ponkanicola*, *L. pseudotheobromae*, and *L. theobromae* are associated with diseased tissues from twigs, branches, and trunks showing symptoms including cankers, cracking, dieback, and gummosis. All *Lasiodiplodia* species were pathogenic to *Citrus reticulata* shoots inoculated in vitro [22]. In Egypt, *L. laeliocattleyae*, *L. pseudotheobromae*, and *L. theobromae* were reported from the symptomatic branches of *C. reticulata* and *C. sinensis* exhibiting dieback. Pathogenicity test results showed that all *Lasiodiplodia* species were pathogenic [11]. In Iran, *L. citricola*, *L. gilanensis*, *L. iraniensis*, *L. pseudotheobromae*, and *L. theobromae* have been identified in citrus branches (*Citrus* sp. and *C. aurantifolia*) as causing cankers and dieback symptoms [23,24]; in Mexico, *L. theobromae* was reported to cause dieback of *C. sinensis* [25]. In Oman, the causal agents of dieback and gummosis in *C. aurantifolia*, and *C. sinensis* were *L. hormozganensis*, and *L. theobromae*; in *C. reticulata,* it was *L. iraniensis* [26]; and, in the United States of America, *L. iraniensis*, and *L. parva* have been reported as causing gummosis and dieback in *C. sinensis*, and *Citrus* sp., respectively [13,27].

Worldwide, the studies on dieback caused by *Lasiodiplodia* in citrus have not focused on Persian lime. Only one study in Mexico found that the disease agents were *L. brasiliense*, *L. citricola*, *L. pseudotheobromae*, *L. subglobosa*, and *L. theobromae* [28]. However, this study used only the ITS, *tef-1α*, and *tub2* regions. Currently, for species of the genus *Lasiodiplodia*, the use of ITS, *tef-1α*, *tub2*, and *rpb2* results in a more reliable species-level resolution [10,15]. Therefore, some identification errors of the species reported for Persian lime might have occurred.

In the past five years, dieback of Persian lime caused by *Lasiodiplodia* has not been studied. Therefore, it is important to understand the current status of *Lasiodiplodia* species causing dieback in Persian lime. Moreover, in southern Mexico, specifically in the state of Tabasco, the etiological agents have not been determined; this state contributes over 87 thousand tons to Persian lime production [2]. The objectives of this study were to (i) identify *Lasiodiplodia* species associated with dieback of Persian lime in southern Mexico, (ii) compare the previously described species associated with dieback in Persian lime with the current state of the species in the region, using the four recommended molecular markers, and (iii) evaluate their pathogenicity and virulence in excised green shoots and certified nursery plants of Persian lime.

## 2. Materials and Methods

### 2.1. Field Survey and Sampling

**Figure 1:**
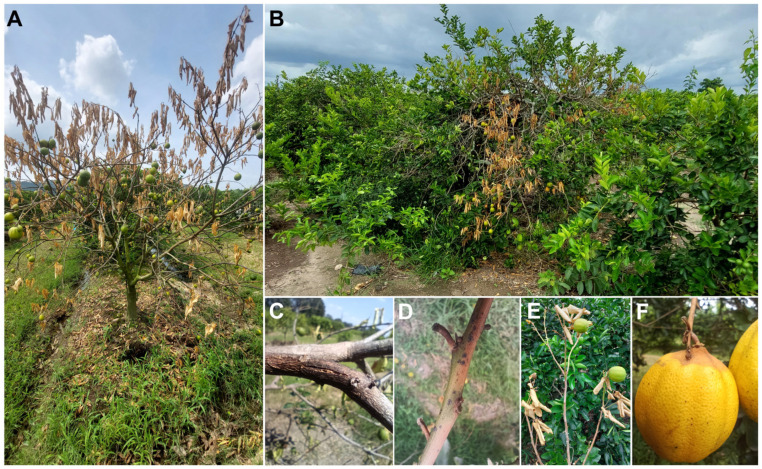
Symptoms caused by *Lasiodiplodia* spp. on *Citrus latifolia* (Persian lime) in southern Mexico. (**A**) Tree showing defoliation and tree decline. (**B**) Tree exhibiting dieback of twigs and branches. (**C**) Sunken canker on branch. (**D**) Twig dieback. (**E**) Branch dieback. (**F**) Fruit mummification.

During June, July, and August of 2023, a survey was conducted of 230 hectares of Persian lime in the main producing region of Tabasco, Mexico. A total of thirty symptomatic plant tissues showing canker, gummosis, and branch dieback were collected (Figure 1), utilizing a completely random sampling method for symptomatic trees. The plant tissue was stored in marked plastic bags and placed in a plastic container with ice for transport to the laboratory.

### 2.2. Fungal Isolations from Persian Lime Branches with Dieback

Fungal isolates were obtained following standard protocol [28]. Fragments of approximately 3 cm from each branch were cut from the margin between the necrotic and healthy tissue zones. These were placed into a 50 mL conical tube containing 20 mL of water plus 5% commercial powdered detergent for 10 min to remove dirt and insects, then immersed in 0.6% sodium hypochlorite for 1 min, rinsed three times with sterile water, and blotted dried on sterile paper. Five pieces of wood (approximately 5 mm^2^ each) were placed into 100-by-15 mm Petri dishes containing potato dextrose agar (PDA; Difco, Detroit, MI, USA, 49 g L^−1^) supplemented with 0.5 g L^−1^ streptomycin sulfate and 0.4 g L^−1^ penicillin (Sigma-Aldrich Co., St. Louis, MO, USA). Plates were incubated at 25 °C for 48 h in the dark. 

Selected emerging fungal colonies were transferred to Petri dishes containing 2% water agar, incubated in the dark for 48 h, and purified by transferring hyphal tips to Petri dishes containing PDA and incubated at 25 °C in the dark. The isolates used in this study were stored at −80 °C in 15% glycerol and deposited in the Culture Collection of Phytopathogenic Fungi of the Phytosanitary Diagnosis Laboratory of the Ixtacuaco Experimental Field of the National Institute of Forestry, Agricultural, and Livestock Research (INIFAP), where they are available upon request (https://www.gob.mx/inifap, accessed on 1 July 2024).

### 2.3. DNA Extraction, Polymerase Chain Reaction Amplification, and Sequencing

Isolates were cultured on PDA and incubated at 25 °C for 7 days. Aerial mycelium was directly collected from the medium using a sterile scalpel blade and transferred into 2 mL microtubes. Total genomic DNA was extracted using the cetyl trimethylammonium bromide (CTAB) method with slight modifications [29]. DNA concentrations were quantified using a NanoDrop OneC (Thermo Fisher Scientific, Madison, WI, USA), the DNA samples were diluted to a concentration of 100 ng/µL.

Partial regions of four loci, the internal transcribed spacer region (ITS), the β-tubulin gene (*tub2*), the translation elongation factor 1-alpha gene (*tef1-α*), and the DNA-directed RNA polymerase II second largest subunit (*rpb2*) were amplified using specific primer sets (Table 1).

All amplification reactions were performed in a total 25 μL volume mixture consisting of 12.5 μL of BlasTaq 2X PCR MasterMix (Applied Biological Materials, Vancouver, BC, Canada), 9.5 μL of Water Molecular Biology, 1 μL of each forward and reverse primer at a concentration of 10 μM, and 1 μL of 100 ng/μL DNA template. The amplification conditions comprised an initial denaturation step at 95 °C for 3 min, followed by 35 cycles of denaturation at 95 °C for 15 s, annealing at 55 °C for ITS region, 58 °C for *tef1-α* gene, and 60 °C for *tub2* and *rpb2* genes for 15 s, and extension at 72 °C for 10 s, followed by a final extension at 72 °C for 5 min. The PCR assays were conducted in a MiniAmp plus thermocycler (Thermo Fisher Scientific, Madison, WI, USA). The PCR products were separated by electrophoresis in a 1.5% agarose gel at 60 V for 90 min stained with ethidium bromide. The amplified PCR products were purified using Wizard SV Gel and PCR Clean-Up System (Promega, Madison, WI, USA) and sequenced in both directions by LANBAMA Laboratory (IPICYT, SLP, San Luis Potosi, Mexico), using the Sanger method.

### 2.4. Phylogenetic Analyses

Forward and reverse sequences were assembled using the Staden Package [34]. Sequences of each of the ITS, *tef1-α*, *tub2*, and *rpb2* loci from 36 well-documented extype *Lasiodiplodia* species from culture [15] were retrieved from GenBank and aligned with sequences of the isolates obtained in this study (Table 2) using the MAFFT v.7 sequence alignment program [35]. The alignments were then manually checked and edited using MEGA XI [36]. Subsequently, the alignment of each locus was loaded into SequenceMatrix v.1.8 [37] to construct the concatenated matrix.

The phylogenetic trees for each locus (ITS, *tef1-α*, *tub2*, and *rpb2*) and for the concatenated matrix were inferred using both maximum likelihood (ML) and Bayesian inference (BI) criteria. ModelTest-NG v.0.1.7 [38] was employed to select evolutionary models independently for each locus and for all loci under the Akaike information criterion (AIC) in both BI and ML analyses. 

ML analyses were performed using RAxML-HPC2 [39], with nonparametric bootstrap iterations run for 1000 replications employing the GTR+G+I substitution model. BI was conducted using MrBayes on XSEDE (v.3.2.7a) [40], implemented on the CIPRES Science Gateway portal (www.phylo.org, accessed on 1 July 2024) [41]. The BI trees were constructed utilizing the Markov chain Monte Carlo (MCMC) algorithm with four runs and four chains per run, running 10,000,000 generations. Trees and parameter values were sampled every 1000 generations, resulting in 10,000 trees. The initial 2500 trees were discarded as the burn-in phase, and the remaining 7500 trees were used to calculate the posterior probabilities (PPs) in the majority rule consensus tree. Tree topologies were visualized using the FigTree v1.4.0 program [42]. Sequences generated in this study were deposited in GenBank (Table 2), and the alignments and trees are available from TreeBASE (http://purl.org/phylo/treebase/phylows/study/TB2:S31354, accessed on 1 July 2024).

### 2.5. Pathogenicity Tests on Detached Branches of Persian Lime

The pathogenicity of the fungal strains was evaluated based on their ability to induce necrosis and gummosis in detached shoots collected from symptomless *C. latifolia* trees, following the methods outlined by Adesemoye et al. (2014) and Berraf-Tebbal et al. (2020) [19,27]. Shoots with a diameter of 15 mm and approximately 20 cm in length were selected. They were then surface-disinfected with water containing 5% commercial powdered detergent for 10 min to remove dirt and insects, followed by treatment with 70% ethanol. Subsequently, the shoots were wounded on an intermediate internode using a scalpel.

For each strain, a 5 mm diameter mycelial disk taken from a 7-day-old colony growing on PDA was placed into the wound. Negative controls were inoculated with fresh, noncolonized PDA plugs. The point of inoculation was covered with parafilm to prevent desiccation. The detached branches were then well watered and maintained in a humid chamber under laboratory conditions. Three replicates per isolate were used, and an equal number of detached branches served as controls. One month after inoculation, the lengths of lesions produced by each strain were measured. Necrotic tissue from the margin of the lesions was collected at 30 days after inoculation, placed onto PDA, and molecularly identified to fulfill Koch’s postulates.

### 2.6. Pathogenicity on Persian Lime Plants from Certified Commercial Nursery and Virulence Tests

The pathogenicity of the 12 representative *Lasiodiplodia* isolates identified phylogenetically was tested on healthy 18-month-old Persian lime plants obtained from a certified commercial nursery. The inoculation procedure followed the protocol described by Bautista-Cruz et al. (2019). Each Persian lime plant was wounded 30 cm from the grafting area using a sterile scalpel, and a colonized PDA disk (5 mm diameter) from a 7-day-old culture was placed onto the wound site. The inoculation site was then covered with wet sterile cotton and sealed with parafilm to prevent desiccation. Five plants were inoculated with each isolate, while the control group received noncolonized PDA disks. Immediately after inoculation, each plant was enclosed in a plastic bag sprinkled with sterile distilled water for 72 h to maintain humidity. All plants were kept in a greenhouse under natural light and temperature conditions [28].

Virulence assessments were conducted 30 days after inoculation by removing the bark and measuring the lesion length in the wood using a digital caliper. The experiment was conducted twice to ensure accuracy and reliability. In both experiments, differences in virulence among *Lasiodiplodia* strains were analyzed with a one-way ANOVA and using the minimum significant difference (*p* ≤ 0.05) test with R v.3.5.1 statistical software.

To complete Koch’s postulates in both experiments, necrotic tissue from the margin of the lesions was sampled and plated onto PDA. The recovered fungal isolates were identified by amplifying and sequencing the *tef1-α* region. Since control plants did not display necrosis, cankers, or gummosis symptoms, and *Lasiodiplodia* spp. were not recovered from the mock-inoculated negative controls, it can be inferred that the plants were not latently infected with these pathogens prior to inoculation.

## 3. Results

### 3.1. Sample Collection, Isolation, and DNA Sequencing

Out of the 9229 Persian lime trees inspected across 230 hectares of Persian lime orchards in the state of Tabasco, Mexico, symptoms of gummosis, stem cankers, twig and branch dieback, fruit mummification, and decline (Figure 1) were detected in 4502 trees, representing a disease incidence of 48.78%. A total of 40 fungal isolates were obtained from diseased tissues collected from symptomatic Persian lime trees; cultural variability was observed in terms of the growth and color of each strain (Figure S1).

**Figure 2:**
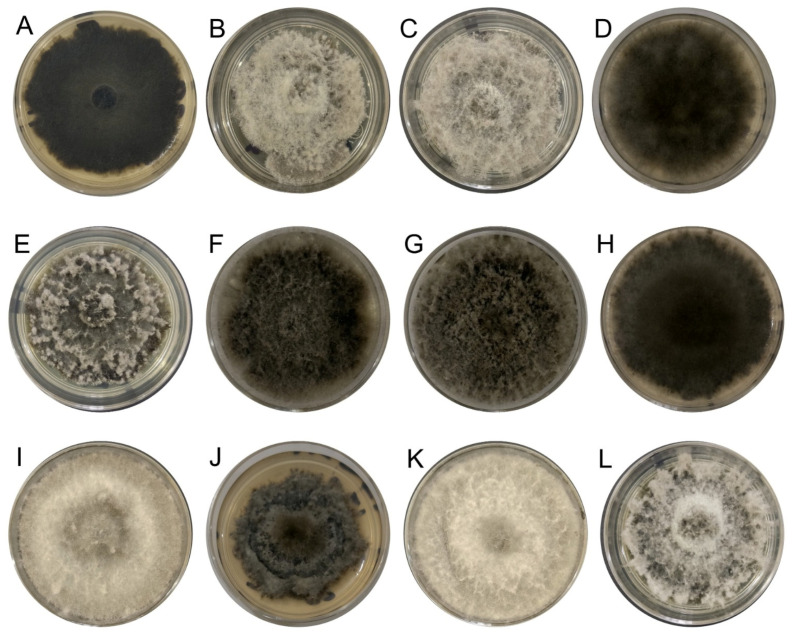
*Lasiodiplodia* culture grown on PDA at 25 °C for 7 days. (**A**) IXBLT3, *L. lignicola*. (**B**) IXBLT4, *L. pseudotheobromae*. (**C**) IXBLT5, *L. pseudotheobromae*. (**D**) IXBLT6, *L. pseudotheobromae*. (**E**) IXBLT7, *L. theobromae*. (**F**) IXBLT9, *L. theobromae*. (**G**) IXBLT10, *L. theobromae*. (**H**) IXBLT12, *L. pseudotheobromae*. (**I**) IXBLT14, *L. iraniensis*. (**J**) IXBLT15, *L. mexicanensis*. (**K**) IXBLT16, *L. iraniensis*. (**L**) IXBLT18, *L. pseudotheobromae*.

The 40 fungal strains obtained from Persian lime plants exhibiting cankers, as well as twig and branch dieback symptoms were identified at the genus level based on BLAST analysis of the ITS region, with 28 identified as *Lasiodiplodia* spp. additionally, their cultural characteristics of growth on PDA were also consistent with those of the *Lasiodiplodia* genus (Figure 2). 

The other 12 isolates belonged to the genera *Diaporthe* (3), *Fusarium* (8), and *Pestalotiopsis* (1) (Figure S1). Derived from the BLAST analysis of the ITS region, the sequences of *tub2*, *tef1-α*, and *rpb2* were obtained for twelve representative *Lasiodiplodia* strains for subsequent phylogenetic analysis.

### 3.2. Phylogenetic Analyses

For the phylogenetic identification of *Lasiodiplodia* species, the combined datasets of four loci, ITS, *tub2*, *tef1-α*, and *rpb2*, comprising 81 *Lasiodiplodia* isolates, including the sequences of the 12 strains from this study, were analyzed alongside 69 sequences of 36 taxa with their extype specimens. *Diplodia seriata* (CBS 112555) was included and used as an outgroup taxon. The GTR+G+I model was selected for the concatenated loci.

**Figure 3:**
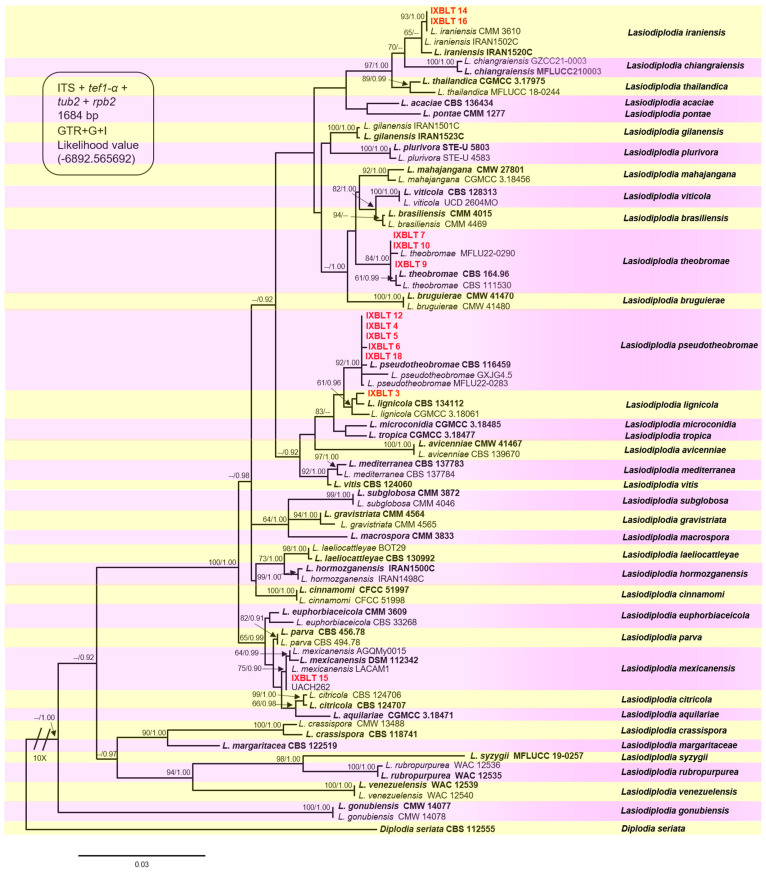
Phylogenetic tree of *Lasiodiplodia* generated from ML analysis of the combined dataset of ITS, *tef1-α*, *tub2*, and *rpb2*. Bootstrap support values for ML ≥ 60% and Bayesian posterior probabilities (PPs) ≥ 0.90 are indicated above at the nodes. Ex−type strains are indicated in bold, and the species are delimited with colored blocks. The isolates collected in the present study are indicated in bold red letters with the nomenclature IXBLT followed by its strain number. The tree was rooted to *Diplodia seriata* (CBS 112555).

The final alignment comprised 1684 characters, including gaps (ITS = 476, *tef1-α* = 324, *tub2* = 394, *rpb2* = 490). Both maximum likelihood (ML) and Bayesian inference (BI) analyses produced trees with similar topologies. The best-scoring ML tree with a final likelihood value of −6892.565692 is presented in Figure 3. The combined datasets for our twelve sequences resulted in the ubication of these strains in five clades, corresponding to the previously described *Lasiodiplodia* species, with moderate to high bootstrap supports and high posterior probabilities. Strains IXBLT14 and IXBLT16 clustered in the *Lasiodiplodia iraniensis* clade with strain CMM 3610, with a bootstrap support of 93% (ML)/1.00 posterior probability (PP); strains IXBLT7, IXBLT9, and IXBLT10 clustered in the *Lasiodiplodia theobromae* clade with *L. theobromae* CBS 164.96, CBS 111530, and MFLU22-0290, with an 84% ML/1.00 PP. The third clade comprised five strains: IXBLT4, IXBLT5, IXBLT6, IXBLT12, and IXBLT18, grouped with *Lasiodiplodia pseudotheobromae* CBS 116459, GXJG4.5, and MFLU22-0283 with 92% ML/1.00 PP. In the fourth clade, only IXBLT3 clustered with *Lasiodiplodia lignicola* (CBS 134112 and CGMCC 3.18061) with a 61% ML/0.96 PP; finally, IXBLT15 clustered in the *Lasiodiplodia mexicanensis* clade (LACAM1, AGQMy0015, and DSM 112342) with a 75% ML/0.90 PP. Furthermore, regarding the species *L. citricola* of Persian lime from Mexico, our findings demonstrated that strain UACH262, previously identified as *L. citricola* [28], is actually *L. mexicanensis* (Figure 3). 

Therefore, in Persian lime trees, *L. pseudotheobromae* was the most frequently isolated species (41.6%), followed by *L. theobromae* (25%), *L. iraniensis* (16.6%), *L. lignicola* (8.3%), and *L. mexicanensis* (8.3%).

### 3.3. Pathogenicity and Virulence on Detached Branches and Plants from Certified Commercial Nursery of Persian Lime

**Figure 4:**
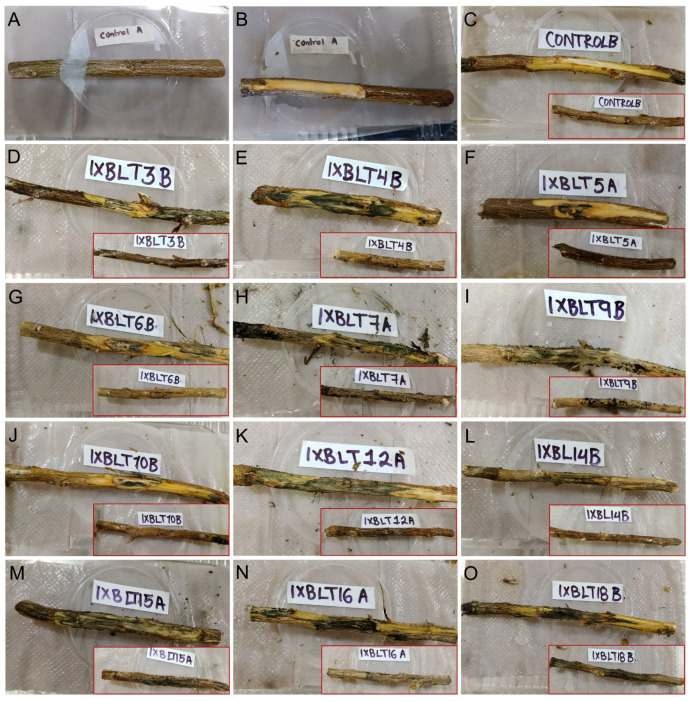
Pathogenicity test on detached branches of Persian lime. (**A**–**C**) Negative controls inoculated with fresh, noncolonized PDA plugs. (**D**) IXBLT3 strain of *L. lignicola*. (**E**–**G**) IXBLT4, IXBLT5, IXBLT6 strains of *L. pseudotheobromae*. (**H**–**J**) IXBLT7, IXBLT9, IXBLT10 strains of *L. theobromae*. (**K**) IXBLT12 strain of *L. pseudotheobromae*. (**L**) IXBLT14 strain of *L. iraniensis*. (**M**) IXBLT15 strain of *L. mexicanensis*. (**N**) IXBLT16 strain of *L. iraniensis*. (**O**) IXBLT18 strain of *L. pseudotheobromae*. Red boxes show the detached branch before the bark was removed.

Koch’s postulates for the *Lasiodiplodia* strains obtained from Persian lime tissue with dieback were completely corroborated by inoculating disks of PDA with mycelium on detached branches and certified plants. Thirty days after inoculation, all of the isolates belonging to the five *Lasiodiplodia* species identified in this study were pathogenic to Persian lime, with different degrees of severity, which was not the case for species belonging to other fungal genera (Figure S2). The wood from detached branches exhibited necrotic lesions that extended from both sides of the point of inoculation (Figure 4).

**Figure 5:**
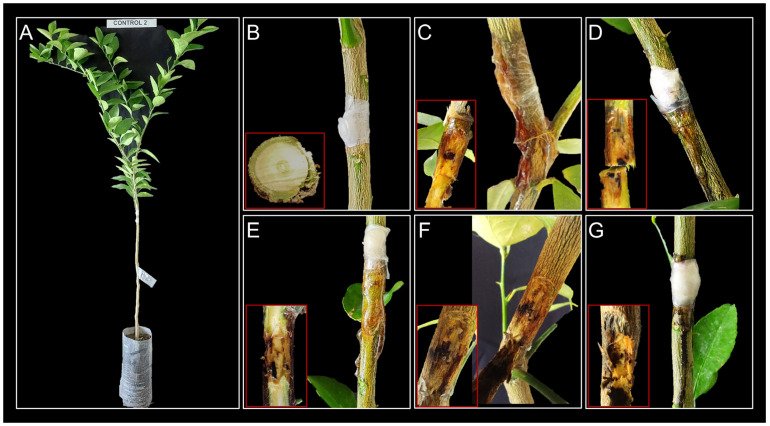
Pathogenicity test on 18-month-old Persian lime plants from certified nursery. (**A**,**B**) Negative controls inoculated with fresh, noncolonized PDA plugs. (**C**) IXBLT3 strain of *L. lignicola*. (**D**) IXBLT9 strain of *L. theobromae*. (**E**) IXBLT12 strain of *L. pseudotheobromae*. (**F**) IXBLT14 of *L. iraniensis*. (**G**) IXBLT15 of *L. mexicanensis*. Red boxes show the stem with the bark removed.

On Persian lime plants, the *Lasiodiplodia* strains induced the formation of gum exudations and necrosis in the tissue upward and downward from the point of inoculation (Figure 5). In both cases, the control plants showed no signs of the disease. *Lasiodiplodia* strains were consistently recovered from affected branches, while none were isolated from healthy control plants, thus satisfying Koch’s postulates.

**Figure 6:**
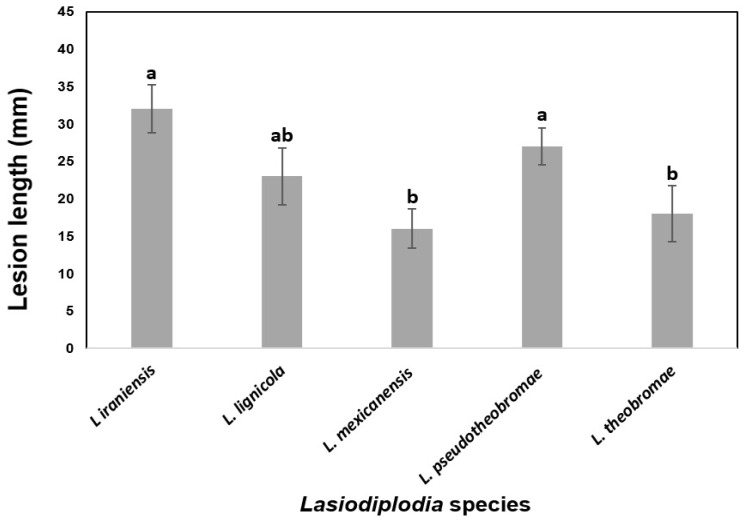
Virulence of five *Lasiodiplodia* species associated with dieback of Persian lime as measured by mean internal lesion lengths (millimeters). Data are lesion sizes measured 30 days after inoculation with mycelium-colonized agar plugs inserted into wounded stem of 18-month-old Persian lime plants from certified nursery. Bars above columns are the standard errors of the means. Columns with the same letter do not differ significantly according to MSD test (*p* ≤ 0.05).

To determine the virulence, the lesion lengths caused by the most aggressive strain of each *Lasiodiplodia* species from two independent experiments on certified nursery Persian lime plants were averaged (Figure 6). There were significant differences in internal necrosis length produced by the different *Lasiodiplodia* species (*p* < 0.05). The longest mean lesions were produced by *L. iraniensis*, followed by *L. pseudotheobromae* and *L. lignicola*, which were the most virulent species. On the other hand, shorter mean lesions were induced by *L. theobromae* and *L. mexicanensis*, which were considered the least virulent species.

## 4. Discussion

The present study is the first to investigate the occurrence and pathogenicity of fungal species associated with twig and branch dieback, cankers, and decline of Persian lime trees (*Citrus latifolia*) in southern Mexico. Moreover, we elucidated the current status of *Lasiodiplodia* species causing disease in Mexico. Through multilocus molecular phylogenetic identification, pathogenicity, and virulence tests, five species were identified: *L. pseudotheobromae*, *L. theobromae*, *L. iraniensis*, *L. lignicola*, and *L. mexicanensis*. The latter two species are reported for the first time as causal agents of the disease in citrus.

The earliest reports characterizing the causal agents of citrus dieback date back to the 1900s. For orange (*C. sinensis*), *Diplodia natalensis* (family Botryosphaeriaceae) was identified as the causal agent of dieback, a disease known as “gummosis induction” [43]. In Robinson tangerine (*C. reticulata*), the causal agent of branch dieback was identified as *L. theobromae* [44]. However, the molecular characterization of fungi was not possible at that time. Currently, there are numerous reports describing the causal agents of dieback, cankers, gummosis, and decline in various citrus species [11,13,22,23,24,25,26,27,28].

In this study, 30 symptomatic branches of Persian lime were collected, and 40 fungal isolates were obtained. Based on their cultural growth characteristics on PDA and BLAST analysis of the ITS region, the isolates belong to the genera *Diaporthe* (7.5%), *Fusarium* (20%), *Lasiodiplodia* (70%), and *Pestalotiopsis* (2.5%), (Figure S1). It is not unusual to isolate other fungal genera from tissues exhibiting symptoms of twig and branch dieback, cankers, and fruit rot. In other studies, in addition to *Lasiodiplodia*, fungi from the genera *Alternaria*, *Cladosporium*, *Colletotrichum*, *Cyphellophora*, *Curvularia*, *Diplodia*, *Dothiorella*, *Eutypella*, *Fusarium*, *Geotrichum*, *Neofusicoccum*, *Neoscytalidium*, *Nigrospora*, and *Phomopsis* have been isolated [25,27,45,46,47]. The 70% of the isolates belonging to *Lasiodiplodia* is consistent with the findings of previous reports indicating that *Lasiodiplodia* is common in citrus, accounting for 55 to 80% of total isolates [13,19,22,27,46].

Multilocus molecular phylogenetic identification of the fungal isolates, based on combined ITS, *tub2*, *tef1-α*, and *rpb2* sequence datasets, revealed that five *Lasiodiplodia* species were isolated from twig and branch dieback. These species included *L. iraniensis*, *L. lignicola*, *L. mexicanensis*, *L. pseudotheobromae*, and *L. theobromae* (Figure 3). Previously, Bautista-Cruz et al. (2019) reported six *Lasiodiplodia* species causing cankers and dieback in Persian lime: *L. brasiliense*, *L. citricola*, *L. iraniensis*, *L. pseudotheobromae*, *L. subglobosa*, and *L. theobromae*, three of which overlap with the species identified in the present study [28]. Nevertheless, *L. lignicola* and *L. mexicanensis* have not been reported as causing twig and branch dieback, cankers, fruit rot, gummosis, and tree decline in any citrus species, making this the first report of their association with Persian lime, representing a novel host–pathogen interaction.

*L. pseudotheobromae* was the most frequently isolated species (41.6%), which has been reported as causing twig and branch dieback, cankers, and gummosis in various citrus species: in China, on *C. limon*, *C. reticulata*, *C. sinensis*, and *C. unshiu*, being the second most frequent [22]; in Egypt, on *C. sinensis* [11]; in Iran on *Citrus* sp. [23]; in Mexico, on *C. latifolia*, being the most abundant species, consistent with our results [28]; in Pakistan, on *C. reticulata* [48]; in Suriname, from *C. aurantium* [31]; and, in Turkey, on *C. limon* [49].

*L. theobromae* was the second most abundant species isolated from *C. latifolia* (25%); this species has a cosmopolitan distribution, causing a variety of diseases on a wide range of host plants [50]. In citrus, it has been reported as causing twig and branch dieback, cankers, and gummosis: in Chile, on *C. limon* [51]; in China, on *C. grandis* [52], *C. reticulata*, and *C. sinensis* [22]; in Egypt, on *C. reticulata* [11]; in Iran, on *C. aurantifolia* [23]; in Malta, on *C. sinensis* [53]; in Mexico, on *C. latifolia* [28], *C. limon*, *C. paradisi*, and *C. sinensis* without pathogenicity testing [54]; in Oman, on *C. aurantifolia*, *C. reticulata*, and *C. sinensis* [26]; in the USA, isolated from *Citrus* sp. [46]; and, in Venezuela, on *C. limon*, *C. paradisi*, and *C. sinensis* [55].

*L. iraniensis* was the third most common species among the isolates examined in our study (16.6%). This species has previously been reported as a pathogen of *Citrus* sp. in Iran [23], on *C. latifolia* in Mexico [28], on *C. reticulata* in Pakistan [56], and recently on *C. sinensis* in the USA [13]. Therefore, research on this species is consistently growing.

In this work, *L. lignicola* and *L. mexicanensis* were the least commonly isolated species from symptomatic Persian lime tissues. *L. lignicola* was initially discovered as saprobic on the dead wood of an unidentified plant in Thailand, where it was named *Auerswaldia lignicola* [17]. However, phylogenetic studies reclassified it as *Lasiodiplodia*, forming a basal clade for other species [5], and it was also detected in a human keratitis case in a 32-year-old Indian male carpenter in India, in 2012, after trauma caused by a wooden piece [57]. Additionally, it was isolated as an endophytic fungus from the healthy tissue of *Aquilaria crassna* in Laos, suggesting a cosmopolitan role for *L. lignicola* [58]. Recently, *L. lignicola* was identified as causing canker and dieback diseases on *Vangueria infausta* subsp. *rotundata* and *Berchemia discolor* in lower eastern Kenya [59]. Therefore, this is the first report in the world of *L. lignicola* being associated with dieback symptoms in citrus species.

In the present study, we report for the first time that *L. mexicanensis* is a causal agent of canker and dieback in Persian lime. Additionally, we analyzed the current status of *L. citricola* as a causal agent of dieback in Persian lime in Mexico. Our findings clearly demonstrate that strain UACH262, previously identified as *L. citricola* [28], is actually *L. mexicanensis* (Figure 3). In this regard, the existence of hybrids between *L. parva* and *L. citricola* was previously hypothesized, previously suggested for *Lasiodiplodia* sp. LACAM1 obtained from *Mangifera indica* in Peru [60], therefore suggesting that strain UACH262 could be a hybrid, as it groups as a sister clade to *L. citricola* with high bootstrap/posterior probability (100/0.98). However, *Lasiodiplodia* sp. LACAM1 was recently identified as *L. mexicanensis*, a species closely related to *L. parva* and *L. citricola*, differing by a few nucleotides in the ITS, *tub2*, *tef1-α*, and *rpb2* sequences, discarding the hypothesis of LACAM1 being a hybrid [61]. According to Cracraft’s phylogenetic species concept, this approach does not use data on reproductive isolation, such as hybridization, for the recognition of species taxa; in addition, biogeographic history is important [62]. Taking this principle into account, *L. citricola* was first isolated from *Citrus* sp. in Iran in 2010 [23], later from *Juglans regia* [63] and *Prunus dulcis* [64] in the USA, from *Acacia* spp. [65] and *Persea americana* [66] in Italy, and recently from *Eriobotrya japonica*, *Malus domestica*, *Vitis vinifera*, and *Juglans regia* in China [16]. Therefore, phylogenetic and biogeographic data support *L. mexicanensis* as a species distinct from *L. citricola*.

At the nucleotide level, strain UACH262 has the following similarities with the ITS, *tub2*, and *tef1-α* sequences: 100% (533/533), 100% (429/429), and 99.78% (444/445) with the ex-type of *L. mexicanensis*, and 99.79% (476/477), 99.48% (379/381), and 99.02% (302/305) with the extype of *L. citricola*, respectively. The *rpb2* sequences are not available for the UACH262 strain.

On the other hand, in the state of Morelos, Mexico, *L. citricola* was described as a causal agent of dieback in *C. latifolia* [67], but, in that study, only the ITS region (KY271187) was used, presenting 100% (540/540) coverage and identity with the extype of *L. mexicanensis* and 99.79% (476/477) coverage and identity with the extype of *L. citricola*, respectively. Currently, for the accurate identification of *Lasiodiplodia* species, the combination of four loci, ITS, *tef1-α*, *tub2*, and *rpb2*, is necessary for reliable resolution [15]. Therefore, it can be concluded that *L. citricola* has not yet been described as associated with canker and dieback in Persian lime (*C. latifolia*) at this time.

The results of pathogenicity testing showed that the isolates of *L. iraniensis* were the most virulent, causing the formation of gum exudates and necrosis in the tissue (Figure 5F). These findings are consistent with those of Bautista-Cruz et al. (2019), where *L. iraniensis* exhibited the highest virulence along with *L. subglobosa* [28], and Piattino et al. (2024), where *L. iraniensis* isolates produced the largest necrotic areas compared to *Diaporthe* spp. [13]. *L. pseudotheobromae* was the second most virulent in Persian lime (Figure 5E), agreeing with results reported by Bautista-Cruz et al. (2019) [22] and Xiao et al. (2021) [28], where it was one of the most aggressive species on citrus shoots. *L. lignicola* was the third most aggressive species (Figure 5C), with lesion lengths of 23 ± 3.8 mm (Figure 6). This species has typically been characterized as a saprophyte [17] or endophyte [58]. However, pathogenicity tests on *Berchemia discolor* and *Olea europaea* showed lesion lengths of 22.6 ± 3.0 mm and 20.1 ± 2.6 mm, respectively [59], which align with the findings of the present study. Finally, *L. mexicanensis* and *L. theobromae* were the least virulent species (Figure 6), consistent with Bautista-Cruz et al. (2019), where strain UACH262 and *L. theobromae* were the least virulent [28], and with El-Ganainy et al. (2022), where *L. theobromae* showed less severity than *L. pseudotheobromae* and *L. laeliocattleyae* on *Citrus* sp. [11], but contrasting Espargham et al. (2020) [24], where *L. theobromae* was more virulent on *C. aurantifolia* shoots than other fungal species.

## 5. Conclusions

The results presented in this study demonstrate that in southern Mexico, at least five species of the genus *Lasiodiplodia* are responsible for dieback in Persian lime. The identified species were *L. pseudotheobromae*, *L. theobromae*, *L. iraniensis*, *L. lignicola*, and *L. mexicanensis*. The most abundant species was *L. pseudotheobromae*, which was also the most virulent along with *L. iraniensis*. On the other hand, multilocus phylogenetic analyses allowed the identification for the first time that the species *L. lignicola* and *L. mexicanensis* are also responsible for dieback in Persian lime. Additionally, it was determined that the strain previously classified as *L. citricola* actually corresponds to *L. mexicanensis*, confirming that this species causes dieback in Persian lime.

## Figures and Tables

**Table 1:** Sequences of primers used in the identification of *Lasiodiplodia* strains.

Locus	Primer	Sequence	Reference
Internal transcribed spacer (ITS)	ITS5	GGAAGTAAAAGTCGTAACAAGG	[30]
	ITS4	TCCTCCGCTTATTGATATGC	
β-tubulin (*tub2*)	Bt2a	GGTAACCAAATCGGTGCTGCTTTC	[31]
	Bt2b	ACCCTCAGTGTAGTGACCCTTGGC	
Translation elongation factor 1-alpha (*tef1-α*)	EF1-688F	CGGTCACTTGATCTACAAGTGC	[32]
	EF1-1251R	CCTCGAACTCACCAGTACCG	[33]
RNA polymerase II second largest subunit (*rpb2*)	RPB2-5F	GAYGAYMGWGATCAYTTYGG	
	RPB2-7cR	CCCATRGCTTGYTTRCCCAT	

**Table 2:** Culture accession numbers, host, location, and GenBank accession numbers of *Lasiodiplodia* isolates used in the phylogenetic analysis.

Species	Strain	Host	Location	GenBank Accession Number			
				ITS	*tef1-α*	*tub2*	*rpb2*
*Lasiodiplodia acaciae*	CBS 136434 ^T^	*Acacia* sp.	Indonesia	MT587421	MT592133	MT592613	MT592307
*L. aquilariae*	CGMCC 3.18471 ^T^	*Aquilaria* *crassna*	Laos	KY783442	KY848600	N/A	KY848562
*L. avicenniae*	CMW 41467 ^T^	*Avicennia* *marina*	South Africa	KP860835	KP860680	KP860758	KU587878
*L. avicenniae*	CBS 139670	*Avicennia* *marina*	South Africa	KU587957	KU587947	KU587868	KU587880
*L. brasiliensis*	CMM 4015 ^T^	*Mangifera* *indica*	Brazil	JX464063	JX464049	N/A	N/A
*L. brasiliensis*	CMM 4469	*Anacardium* *occidentale*	Brazil	KT325574	KT325580	N/A	N/A
*L. bruguierae*	CMW 41470 ^T^	*Bruguiera gymnorrhiza*	South Africa	KP860832	KP860677	KP860755	KU587875
*L. bruguierae*	CMW 42480	*Bruguiera* *gymnorrhiza*	South Africa	KP860834	KP860679	KP860757	KU587876
*L. chiangraiensis*	MFLUCC21- 0003 ^T^	Unknown host	Thailand	MW760854	MW815630	MW815628	N/A
*L. chiangraiensis*	GZCC21- 0003	Unknown host	Thailand	MW760853	MW815629	MW815627	N/A
*L. cinnamomi*	CFCC 51997 ^T^	*Cinnamomum* *camphora*	China	MG866028	MH236799	MH236797	MH236801
*L. cinnamomi*	CFCC 51998	*Cinnamomum camphora*	China	MG866029	MH236800	MH236798	MH236802
*L. citricola*	CBS 124707 ^T^	*Citrus* sp.	Iran	GU945354	GU945340	KU887505	KU696351
*L. citricola*	CBS 124706	*Citrus* sp.	Iran	GU945353	GU945339	KU887504	KU696350
*L. citricola*	UACH262	*Citrus latifolia*	Mexico	MH277948	MH286541	MH279934	N/A
*L. crassispora*	CBS 118741 ^T^	*Santalum* *album*	Australia	DQ103550	DQ103557	KU887506	KU696353
*L. crassispora*	CMW 13488	*Eucalyptus* *urophylla*	Venezuela	DQ103552	DQ103559	KU887507	KU696352
*L. euphorbiaceicola*	CMM 3609 ^T^	*Jatropha curcas*	Brazil	KF234543	KF226689	KF254926	N/A
*L. euphorbiaceicola*	CMW 33268	*Adansonia* sp.	Senegal	KU887131	KU887008	KU887430	KU887367
*L. gilanensis*	IRAN1523 C^T^	*Citrus* sp.	Iran	GU945351	GU945342	KU887511	KP872462
*L. gilanensis*	IRAN1501C	*Citrus* sp.	Iran	GU945352	GU945341	KU887510	KP872463
*L. gonubiensis*	CMW 14077 ^T^	*Syzygium cordatum*	South Africa	AY639595	DQ103566	DQ458860	N/A
*L. gonubiensis*	CMW 14078	*Syzygium* *cordatum*	South Africa	AY639594	DQ103567	EU673126	N/A
*L. gravistriata*	CMM 4564 ^T^	*Anacardium* *humile*	Brazil	KT250949	KT250950	N/A	N/A
*L. gravistriata*	CMM 4565	*Anacardium* *humile*	Brazil	KT250947	KT266812	N/A	N/A
*L. hormozganensis*	IRAN1500C^T^	*Olea* sp.	Iran	GU945355	GU945343	KU887515	KP872466
*L. hormozganensis*	IRAN1498C	*Mangifera indica*	Iran	GU945356	GU945344	KU887514	KP872467
*L. iraniensis*	IRAN1520C^T^	*Salvadora* *persica*	Iran	GU945348	GU945336	KU887516	KP872468
*L. iraniensis*	IRAN1502C	*Juglans* sp.	Iran	GU945347	GU945335	KU887517	KP872469
*L. iraniensis*	CMM 3610	*Jatropha curcas*	Brazil	KF234544	KF226690	KF254927	N/A
***L. iraniensis***	**IXBLT 14**	***Citrus latifolia***	**Mexico**	**PP778685**	**PP779539**	**PP769242**	**PP784203**
***L. iraniensis***	**IXBLT 16**	***Citrus latifolia***	**Mexico**	**PP778687**	**PP779541**	**PP769244**	**PP784205**
*L. laeliocattleyae*	CBS 130992 ^T^	*Mangifera indica*	Egypt	NR_120002	KU507454	KU887508	KU696354
*L. laeliocattleyae*	BOT 29	*Mangifera indica*	Egypt	JN814401	JN814428	N/A	N/A
*L. lignicola*	CBS 134112 ^T^	Dead wood	Thailand	JX646797	KU887003	KT852958	KU696364
*L. lignicola*	CGMCC 3.18061	Woody branch	China	NR_152983	KX499927	KX500002	KX499965
***L. lignicola***	**IXBLT 3**	***Citrus latifolia***	**Mexico**	**PP778677**	**PP779531**	**PP769234**	**PP784195**
*L. macrospora*	CMM 3833 ^T^	*Jatropha curcas*	Brazil	NR_147349	KF226718	KF254941	N/A
*L. mahajangana*	CMW 27801 ^T^	*Terminalia* *catappa*	Madagascar	NR_147325	FJ900641	FJ900630	N/A
*L. mahajangana*	CGMCC 3.18456	*Aquilaria crassna*	Laos	KY783437	KY848596	KY848529	KY848557
*L. margaritacea*	CBS 122519 ^T^	*Adansonia gibbosa*	Australia	KT852959	EU144065	KU887520	KU696367
*L. mediterranea*	CBS 137783 ^T^	*Quercus ilex*	Italy	KJ638312	KJ638331	KU887521	KU696368
*L. mediterranea*	CBS 137784	*Vitis vinifera*	Italy	KJ638311	KJ638330	KU887522	KU696369
*L. mexicanensis*	DSM 112342 ^T^	*Chamaedorea seifrizii*	Mexico	MW274151	MW604234	MW604243	MW604222
*L. mexicanensis*	AGQMy0015	*Chamaedorea seifrizii*	Mexico	MW274150	MW604233	MW6042423	MW604221
*L. mexicanensis*	LACAM1	*Mangifera indica*	Peru	KU507469	KU507436	N/A	N/A
***L. mexicanensis***	**IXBLT 15**	***Citrus latifolia***	**Mexico**	**PP778686**	**PP779540**	**PP769243**	**PP784204**
*L. microconidia*	CGMCC 3.18485 ^T^	*Aquilaria* *crassna*	Laos	KY783441	KY848614	N/A	KY848561
*L. parva*	CBS 456.78 ^T^	cassava-field soil	Colombia	EF622083	EF622063	KU887523	KP872477
*L. parva*	CBS 494.78	cassava-field soil	Colombia	EF622084	EF622064	EU673114	KU696373
*L. plurivora*	STE-U 5803 ^T^	*Prunus salicina*	South Africa	EF445362	EF445395	KP872421	KP872479
*L. plurivora*	STE-U 4583	*Vitis vinifera*	South Africa	AY343482	EF445396	KU887525	KU696375
*L. pontae*	CMM 1277 ^T^	*Spondias* *purpurea*	Brazil	KT151794	KT151791	KT151797	N/A
*L. pseudotheobromae*	CBS 116459 ^T^	*Gmelina arborea*	Costa Rica	EF622077	EF622057	EU673111	KU696376
*L. pseudotheobromae*	GXJG4.5	*Macadamia integrifolia*	China	MH487656	MH487655	MH487654	N/A
*L. pseudotheobromae*	MFLU22-0283	*Panicum* sp.	Thailand	OQ123587	OQ509114	OQ509083	N/A
***L. pseudotheobromae***	**IXBLT 4**	***Citrus latifolia***	**Mexico**	**PP778678**	**PP779532**	**PP769235**	**PP784196**
***L. pseudotheobromae***	**IXBLT 5**	***Citrus latifolia***	**Mexico**	**PP778679**	**PP779533**	**PP769236**	**PP784197**
***L. pseudotheobromae***	**IXBLT 6**	***Citrus latifolia***	**Mexico**	**PP778680**	**PP779534**	**PP769237**	**PP784198**
***L. pseudotheobromae***	**IXBLT 12**	***Citrus latifolia***	**Mexico**	**PP778684**	**PP779538**	**PP769241**	**PP784202**
***L. pseudotheobromae***	**IXBLT 18**	***Citrus latifolia***	**Mexico**	**PP778688**	**PP779542**	**PP769245**	**PP784206**
*L. rubropurpurea*	WAC 12535 ^T^	*Eucalyptus grandis*	Australia	DQ103553	DQ103571	EU673136	KP872485
*L. rubropurpurea*	WAC 12536	*Eucalyptus* *grandis*	Australia	DQ103554	DQ103572	KU887530	KP872486
*L. subglobosa*	CMM3872 ^T^	*Jatropha curcas*	Brazil	KF234558	KF226721	KF254942	N/A
*L. subglobosa*	CMM 4046	*Jatropha curcas*	Brazil	KF234560	KF226723	KF254944	N/A
*L. syzygii*	MFLUCC 19-0257 ^T^	*Syzygium* *samarangense*	Thailand	MT990531	MW016943	MW014331	N/A
*L. thailandica*	CGMCC 3.17975 ^T^	*Acacia confusa*	China	KX499879	KX499917	KX499992	KX499955
*L. thailandica*	MFLUCC 18-0244	*Swietenia* *mahagoni*	Thailand	MK347789	MK340870	MK412877	N/A
*L. theobromae*	CBS 164.96 ^T^	*Fruit along coral reef coast*	Papua New Guinea	AY640255	AY640258	KU887532	KU696383
*L. theobromae*	CBS 111530	*Leucospermum* sp.	USA	EF622074	EF622054	KU887531	KU696382
*L. theobromae*	MFLU22-0290	*Artocarpus heterophyllus*	Thailand	OQ123594	OQ509109	OQ509088	OQ509080
***L. theobromae***	**IXBLT 7**	***Citrus latifolia***	**Mexico**	**PP778681**	**PP779535**	**PP769238**	**PP784199**
***L. theobromae***	**IXBLT 9**	***Citrus latifolia***	**Mexico**	**PP778682**	**PP779536**	**PP769239**	**PP784200**
***L. theobromae***	**IXBLT 10**	***Citrus latifolia***	**Mexico**	**PP778683**	**PP779537**	**PP769240**	**PP784201**
*L. tropica*	CGMCC3.18477 ^T^	*Aquilaria crassna*	Laos	KY783454	KY848616	KY848540	KY848574
*L. venezuelensis*	WAC 12539 ^T^	*Acacia mangium*	Venezuela	DQ103547	DQ103568	KU887533	KP872490
*L. venezuelensis*	WAC 12540	*Acacia mangium*	Venezuela	DQ103548	DQ103569	KU887534	KP872491
*L. viticola*	CBS 128313 ^T^	*Vitis vinifera*	USA	HQ288227	HQ288269	HQ288306	KU696385
*L. viticola*	UCD 2604MO	*Vitis vinifera*	USA	HQ288228	HQ288270	HQ288307	KP872493
*L. vitis*	CBS: 124060 ^T^	*Vitis vinifera*	Italy	KX464148	KX464642	KX464917	KX463994
*Diplodia seriata*	CBS 112555 ^T^	*Vitis vinifera*	Portugal	AY259094	AY573220	DQ458856	KX463962

^T^ Extype strains; N/A: sequences not available. Newly generated sequences in this study are in bold. BOT: A. M. Ismail, Plant Pathology Research Institute, Egypt. CBS: Centraalbueau voor Schimmelcultures, Utrecht, The Netherlands. CFCC: China Forestry Culture Collection Center, Beijing, China. CGMCC: China General Microbiological Culture Collection Center. CMM: Culture Collection of Phytopathogenic Fungi ‘Prof. Maria Menezes’ (CMM) at the Universidade Federal Rural de Pernambuco, Brazil. CMW: Tree Pathology Co-operative Program, Forestry and Agricultural Biotechnology Institute, University of Pretoria, South Africa. GZCC: Guizhou Academy of Agricultural Sciences Culture Collection, Guizhou, China. IXBLT: Ixtacuaco Experimental Field Fungal Culture Collection of the INIFAP, Mexico. IRAN: Iranian Fungal Culture Collection, Iranian Research Institute of Plant Protection, Iran. MFLUCC: Mae Fah Luang University Culture Collection, Chiang Rai, Thailand. STE-U: Culture collection of the Department of Plant Pathology, University of Stellenbosch, South Africa. UACH: Culture Collection of Phytopathogenic Fungi of the Department of Agricultural Parasitology at the Chapingo Autonomous University, Mexico. UCD: University of California, Davis, Plant Pathology Department Culture Collection. WAC: Department of Agriculture, Western Australia Plant Pathogen Collection, Australia. ITS: internal transcribed spacer regions; *tef1-α*: translation elongation factor 1-alpha gene; *tub2*: beta-tubulin gene; *rpb2*: DNA-directed RNA polymerase II second largest subunit.

## Data Availability

The data generated are available upon a reasonable requisition.

## Supplementary Materials

The following supporting information can be downloaded at: https://www.mdpi.com/article/10.3390/jof10070484/s1, Figure S1: Culture grown on PDA at 25 °C for 7 days of fungal isolates from Persian lime; Figure S2: *Diaporthe*, *Fusarium*, and *Pestalotiopsis* inoculation on detached branches of Persian lime.
