# Supplementary material for: Phylogenetic and Pathogenic Evidence Reveals Novel Host–Pathogen Interactions between Species of Lasiodiplodia and Citrus latifolia Dieback Disease in Southern Mexico
Source: J Fungi (Basel). 2024 Jul 14;10(7):484. doi: 10.3390/jof10070484 (PMC11278223; doi:10.3390/jof10070484)
Supplement: Supplementary file 1 [file jof-10-00484-s001.zip › jof-3092987-supplementary.pdf]

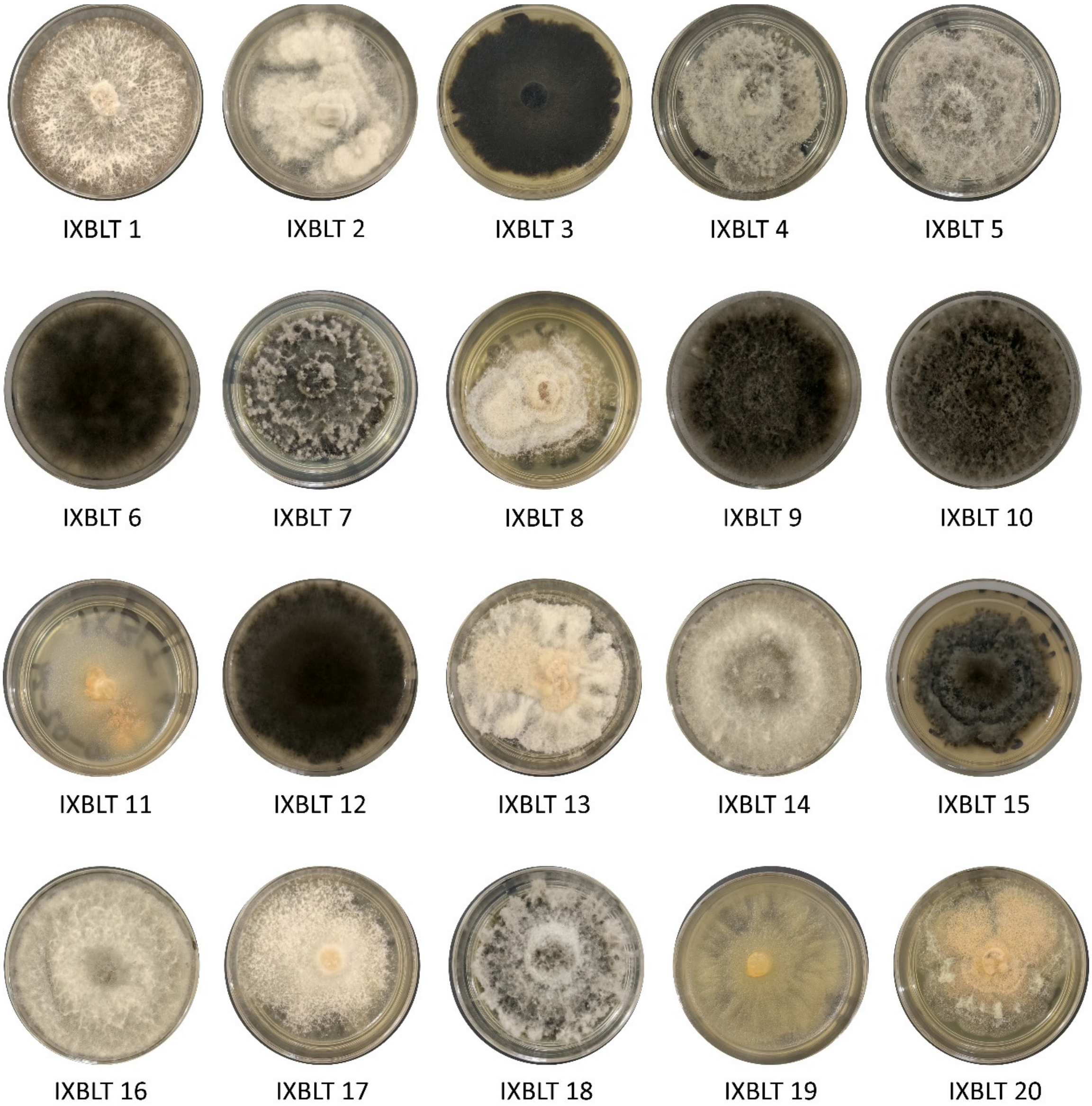

Figure S1. Culture grown on PDA at 25 °C for 7 days of fungal isolated from Persian lime. IXBLT 1= *Fusarium* sp.; IXBLT 2= *Diaporthe* sp.; IXBLT 3= *Lasiodiplodia lignicola*; IXBLT 4= *L. pseudotheobromae*; IXBLT 5= *L. pseudotheobromae*; IXBLT 6= *L. pseudotheobromae*; IXBLT 7= *L. theobromae*; IXBLT 8= *Fusarium* sp. (FIESC); IXBLT 9= *L. theobromae*; IXBLT 10= *L. theobromae*; IXBLT 11= *Fusarium* sp.; IXBLT 12= *L. pseudotheobromae*; IXBLT 13= *Fusarium* sp.; IXBLT 14= *L. iraniensis*; IXBLT 15= *L. mexicanensis*; IXBLT 16= *L. iraniensis*; IXBLT 17= *Fusarium* sp.; IXBLT 18= *L. pseudotheobromae*; IXBLT 19= *Pestalotiopsis* sp.; IXBLT 20= *Diaporthe* sp.

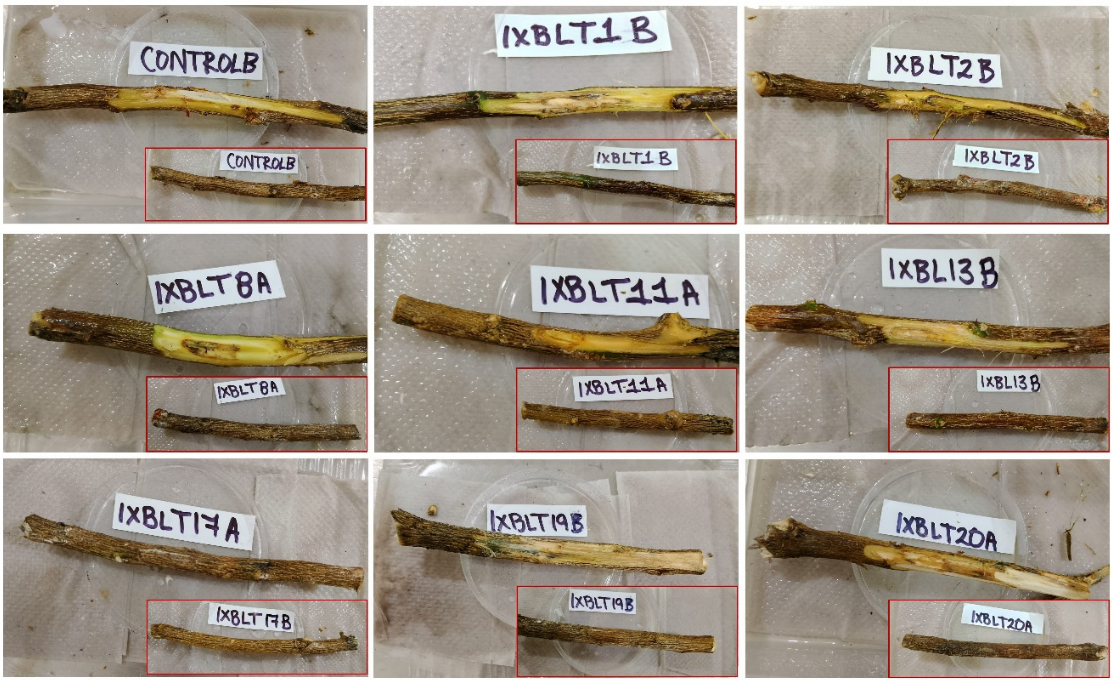

Figure S2. *Diaporthe*, *Fusarium*, and *Pestalotiopsis* inoculation on detached branches of Persian lime. Control B= branch inoculated with fresh, non-colonized PDA plugs; IXBLT 1= *Fusarium* sp.; IXBLT 2= *Diaporthe* sp.; IXBLT 8= *Fusarium* sp. (FIESC); IXBLT 11= *Fusarium* sp.; IXBLT 13= *Fusarium* sp.; IXBLT 17= *Fusarium* sp.; IXBLT 19= *Pestalotiopsis* sp.; IXBLT 20= *Diaporthe* sp. Red boxes show the detached branch before the bark was removed.
